# A Fully Linear Response *G*_0_*W*_0_ Method That Scales Linearly up to Tens of Thousands of Cores

**DOI:** 10.1021/acs.jpca.2c01328

**Published:** 2022-05-18

**Authors:** Paolo Umari

**Affiliations:** † Dipartimento di Fisica e Astronomia, Università di Padova, Padova I-35131, Italy; ‡ CNR-IOM Democritos, Istituto Officina dei Materiali, Consiglio Nazionale delle Ricerchei, Trieste I-34149, Italy

## Abstract

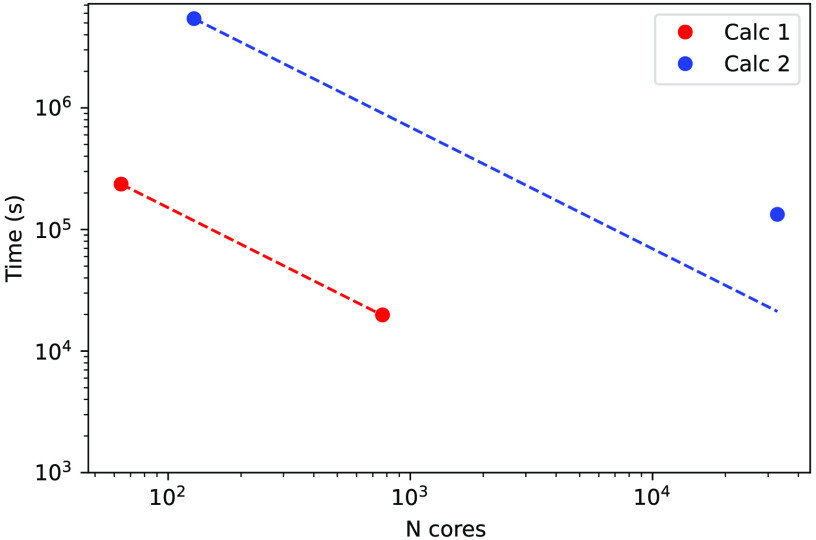

We present a G_0_W_0_ approach that is based on the evaluation of the linear response of the actions of the *G*_0_ and *W*_0_ operators. In this way we avoid sums over empty one-particle orbitals and do not have to explicitly develop the screened Coulomb interaction *W*_0_ on a dedicated basis. For a given orbital, the self-energy is found by summing terms relative to a set of points in the real-space simulation cell. This permits us to easily control the ratio of the accuracy to the computational cost. A trivial parallelization strategy allows strong linear scaling up to tens of thousands of computing cores.

## Introduction

Nowadays, the *GW* approach,^1,2^ which takes its name from the approximation used to write the self-energy operator as a product of the Green’s function *G* with the screened Coulomb interaction *W*, has become a standard tool^3^ for obtaining accurate quasi-particle energies starting from density functional theory (DFT).^4,5^ Despite its popularity, the *GW* approach remains a computationally demanding task, particularly for implementations based on the planewaves–pseudopotentials paradigm.^5^ Unfortunately, this is the framework chosen by a large number of electronic structure codes.^6−10^

The computational burden is determined by the presence, in the formalism, of sums over empty one-particle orbitals and the need to deal with response operators, which must be represented on appropriate, generally large, basis sets. Over the past decade, several groups proposed recipes to alleviate or even remove the problem of the sums over empty orbitals.^9,11−13^ These are based on the same idea of the resolution of the identity, which is at the base of density functional perturbation theory (DFPT).^14^ Indeed, the projector over the conduction manifold can be written as the identity operator minus the projector over the valence manifold.

Importantly, using reduced but *clever* basis sets to represent response operators as either *W* or the polarizability can increase the speed significantly.^15,16^ These approaches require appropriate checks of the quality of the used basis set. This prompted the exploration of other roads as algorithms based on stochastic processes, which led to remarkable increases in speed.^17,18^

Although frequently seen as a postprocessing of DFT calculations, *GW* calculations are much more computational demanding. This calls for elaborate parallelization strategies to take advantage of massively parallel supercomputers.^9,19^

Recently, two research groups presented methods to avoid the computation and memory storage of the entire screened Coulomb interaction *W* within the BSE scheme for the evaluation of neutral excitation energies.^20,21^ Indeed, instead of the operator *W* being calculated, only its action on a vector representing an excitation is determined. This brings the valuable side effect of giving fully converged results, removing any need to check the consistency of the basis for *W*.

Here, we extend the same idea to *G*_0_*W*_0_. Only the action of the screened Coulomb interaction *W*_0_ and that of the Green’s function *G*_0_ on a wave function are needed. These are calculated through ordinary linear response approaches such as those of DFPT. Hence, sums over empty states and dedicated basis sets are avoided altogether. The calculation is cast as a weighted sum over a group of points inside the real-space simulation cell. While using just one grid point yields approximate results (with an average error in the self-energy of ca. 20%), using a few points gives well-converged quasi-particle energies, and adding points permits fully converged results to be reached and used as benchmarks.

Being based on linear response, our method inherits the parallelizations schemes of DFPT codes on one side and is trivially parallelizable over groups of real space points on the other. This means not only that the strong scaling of the code (i.e., keeping the size of the problem fixed) is linear up to tens of thousands of computing cores but also that runs can be easily distributed over a computing grid.

## Method

We limit our discussion to the diagonal *G*_0_*W*_0_ approximation.^2^ Although this procedure avoids self-consistency and identifies the quasi-particle amplitudes with the Kohn–Sham (KS) orbitals, it remains the most popular way to (drastically) improve upon the KS energies.^22^ In a nutshell, the diagonal *G*_0_*W*_0_ scheme prescribes the following self-consistent equation for the quasi-particle energy *E*_*i*_ relative to the *i*th orbital:
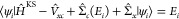
1where operators are indicated with a hat, *Ĥ*^KS^ is the KS Hamiltonian, *V̂*_*xc*_ is the exchange and correlation potential, Σ̂_*x*_ is the (exact) exchange part of the self-energy operator, and Σ̂_*c*_ is the correlation part of the self-energy operator.

The self-energy operator is expressed as the convolution of the DFT Green’s function *G*_0_ with the screened Coulomb interaction *Ŵ*_0_ as follows:
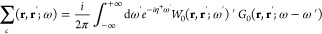
2where η^+^ is a positive infinitesimal, which selects the contour for integration in the Riemann plane. For the dual purpose of avoiding explicit sums over empty orbitals in the calculation of *Ĝ*_0_ and *Ŵ*_0_ and avoiding the introduction of any explicit basis set in the representation of the entirety of *W*_0_, we introduce the following functions for a generic point **r** in the simulation cell:
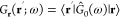
3and
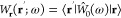
4which should be thought as functions of **r**′ and ω depending on the parameter **r**. Both functions can be calculated through linear response^14,23^ as detailed in the Supporting Information (SI).

We denote the term ⟨ψ_*i*_|Σ̂_*c*_(ω)|**r**⟩, which is obtained through frequency convolution, with the function *S*_*i*_(**r**;ω).
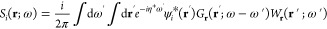
5Note that, in practice, the frequency axis must be discretized. The expectation values of Σ̂_*c*_ read:
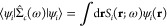
6

To perform an actual computation, this integral requires discretization. In plane-waves codes, wave functions are also available on a grid of equally spaced **r**_α_-points:
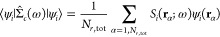
7where the index α runs over all the *N*_*r*,tot_ points of the grid. Summing over all the *N*_*r*,tot_ points would remain a formidable task in general and would be feasible only for tiny systems. To lower the computational burden, we observe that points **r**_α_ for which ψ_*i*_(**r**_α_) ∼ 0 bring a negligible contribution. This calls for a twofold strategy to reduce the number of grid points to be processed. First, we consider a coarser grid that takes, along the three Cartesian directions, one point every *n* of the original dense grid. This leaves us with *N*_tot_/*n*^3^ grid points. We further reduce the number of grid points by imposing the condition |ψ_*i*_(**r**_α_)|>*s*, where *s* is an opportune threshold. We indicate with *N*_*n*,*s*_ the number of grid points to be processed. This yields an approximate value for the self-energy expectation value, which we indicate with ⟨ψ_*i*_|Σ_*c*_(ω)|ψ_*i*_⟩_*n*,*s*_ as follows:
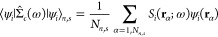
8It is worth noting that the choice *n* = 2 and *s* = 0 gives a fully converged result, as in plane-waves codes it is customary to have real-space grids two-times denser than the corresponding plane-waves ones. As can be easily forseen, ⟨ψ_*i*_|Σ_*c*_(ω)|ψ_*i*_⟩_*n*,*s*_ converges slowly with *N*_*n*,*s*_ such that that the use of eq 8 is impractical.

Convergence can be boosted by inserting weights in the sum over the grid points. We started to observe that if ψ_*i*_ were a reasonable approximation for an eigenstate of Σ̂_*c*_, than just one grid point would give a good estimate of the corresponding expectation value.
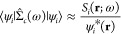
9This relation would become exact if ψ_*i*_ were an eigenstate of Σ̂_*c*_.

In practice, the value depends on the choice of the grid point **r**. To minimize the error, we take the point **r**_*i*_ for which |ψ(**r**_*i*_)| is maximal. The corresponding approximate expectation value ⟨ψ_*i*_|Σ_*c*_(ω)|ψ_*i*_⟩^maxval^ yields
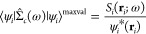
10

It should be kept in mind that ψ_*i*_ can be considered, usually, a reasonable approximation of an eiegenstate of the *G*_0_*W*_0_ Hamiltonian *Ĥ*^KS^ – *V̂*_*xc*_ + Σ̂_*c*_(*E*_*i*_) + Σ̂_*x*_ but not of Σ̂_*c*_. That notwithstanding, this apparently harsh approximation yields values for the expectation values of the self-energy with errors within ca. 20%, as reported in the next section.

This prompted us to generalize the method to the same set of grid points used in eq 8. This was done by weighting the grid points according to ψ_*i*_(**r**_α_). We indicate the results with ⟨ψ_*i*_|Σ_*c*_(ω)|ψ_*i*_⟩_*n*,*s*_^weighted^.
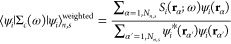
11This is the main result of our work. It is worth noting that if *N*_*n*,*s*_ = 1 we recover the formula of eq 9, while in the opposite limit of a dense grid where *N*_*n*,*s*_ = *N*_*r*,tot_ we recover the same exact results of eq 7. This means that as the number of grid points increases the method tends to fully converged values.

## Results

### Implementation and Validation

Without loosing generality, we chose the analytic continuation scheme^24,25^ where the expectation values of Σ̂_*c*_ are first obtained on the imaginary frequency axis and then analytically continued on the real one. We implemented our method inside the *pw4gww.x* module of the Quantum-Espresso DFT package,^6,26^ which is based on the planewaves–pseudopotentials paradigm. The Brillouin zone is sampled at the sole Γ-point, permitting us to work with real wave functions. For the case of crystalline materials, being limited to the sole Γ-point is not an heavy drawback as we can take advantage of symmetries in real space instead of those in the reciprocal one. Either the Coulomb interaction can be truncated at a given radius, when simulating finite systems, or a proper treatment of periodic boundary conditions is needed.^27^ This requires the long-range elements (head and wings) of the symmetric dielectric matrix, which are calculated through linear response with the *head.x* code. It is worth mentioning that both the DFT charge density and the head and wings of the dielectric matrix can be calculated through arbitrary sampling of the Brillouin zone to achieve convergence. The *pw4gww.x* code generates the *S*_*i*_(**r**_α_;ω) functions. These are then read by a small python program,^28^ which builds the ⟨ψ_*i*_|Σ_*c*_(ω)|ψ_*i*_⟩ expectation values. We have made the code publicly available within Quantum-Espresso.

To illustrate, the capabilities of our method we begin by considering a small molecule, namely methane, as we can easily carry out extensive sets of calculations for it. We address a cubic simulation cell with an edge of 20 Bohr, an energy cutoff of 40 Ry to define the plane-waves basis set representing wave functions, the local density approximation (LDA)^29^ for the DFT exchange and correlation potential, norm-conserving pseudopotentials, and the theoretically optimized structure. Further details regarding the calculations are given in the SI together with the corresponding input files.

**Figure 1:**
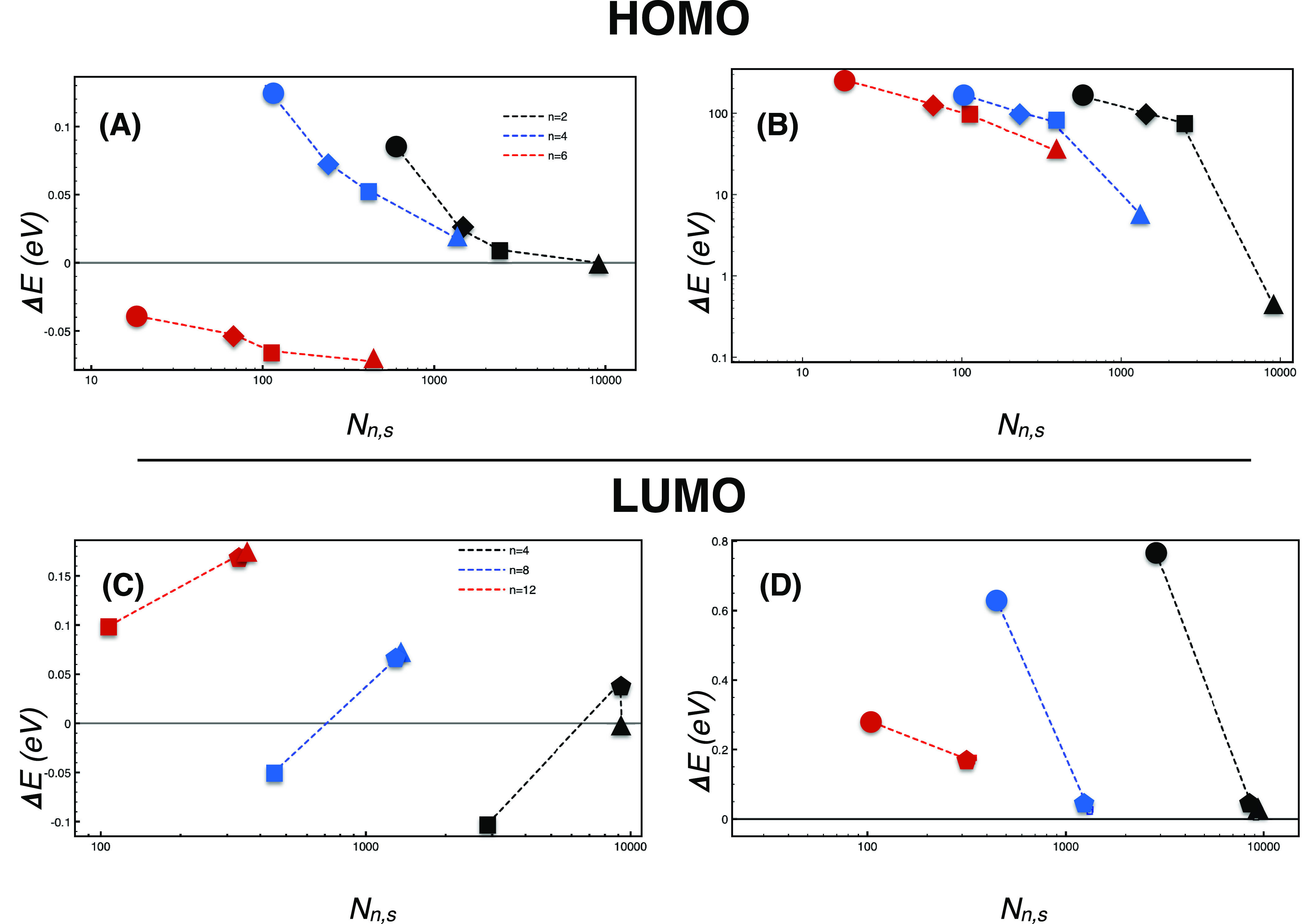
(A and C) Errors and (B and ) absolute errors in the (A and B) HOMO and (C and D) LUMO energies for methane, which were calculated using eq 11 (panel A and C) or eq 8 (panel B and D). The number of real-space points *N*_*n*,*s*_ refers to *n* = 2 (black), *n* = 4 (blue), and *n* = 6 (red) for panels A and B and refers to *n* = 4 (black), *n* = 12 (blue), and *n* = 12 (red) for panels C and D. The threshold *s* was set to 0.1 (triangle), 0.5 (pentagon), 1 (square), 2 (diamond), and 3 (circle).

Errors defined with respect to our most accurate results are displayed in Figure 1. For the HOMO, we could use the same grid as that used for the wave functions (*n* = 2) and *s* values down to *s* = 0.1. This implies 9095 grid points. From panel A we see that our method permits us to choose a larger *n* value and a larger *s* value so that *N*_*n*,*s*_ can be decreased to ∼100 while maintaining the accuracy within 0.1 eV. This means that we can reduce the computational cost by two orders of magnitude. In contrast, the use of the *naif* approach from eq 8 leads to the catastrophic scenario of panel B; apart from the densest grids, all the others yield errors larger than 1 eV. These very large deviations obliged us to use a logarithmic scale in panel B of Figure 1.

For the LUMO, we find a similar situation. In this case the DFT orbital is highly delocalized (see Figure S1), so we considered smoother grids starting with *n* = 4. In this case our method also permits us to increase the values of *n* and *s* while maintaining the accuracy within 0.1 eV with only ∼100 grid points. At the same time, the method of eq 8 leads to much larger errors.

Our calculated vertical ionization energy (i.e., the HOMO energy with a changed sign) for the methane molecule is 14.2 eV, which is in nice agreement with a similar evaluation (14.1 eV),^22^ albeit starting from PBE^30^ instead of LDA, conducted using the WEST code, which also avoids explicit sums over empty states.^9^ The figure is also in agreement with quantum chemistry coupled-cluster calculations at the CCSD(T) level (14.4 eV), although all these theoretical estimates are larger than the reported measurement (13.6 eV).^22^ Our electron affinity energy (i.e., the LUMO energy with a changed sign) is −0.2 eV. Finally, it is worth noting that the use of only one grid-point, such as in eq 10, leads to a relatively small error of 0.4 eV for the HOMO, while that for the LUMO, which is delocalized, is larger at 1.8 eV.

We turn now to bulk silicon, as it is a prototypical crystalline system. As the present implementation of our method is limited to Γ-only sampling, we are obliged to consider supercells. We start with a cubic model of Si at the experimental lattice constant comprised of 64 atoms. The DFT-LDA charge density and the head and wings of the symmetric dielectric matrix are evaluated with a regular 4 × 4 × 4 mesh of *k*-points. Additional details together with input files can be found in the SI. Taking advantage of the symmetry of the edge orbitals, we use grid points belonging to one subcell comprised of eight atoms. In this case, we can easily reach full convergence with either method by setting *n* = 2 and *s* = 0. This requires the evaluation of 3375 grid points.

**Figure 2:**
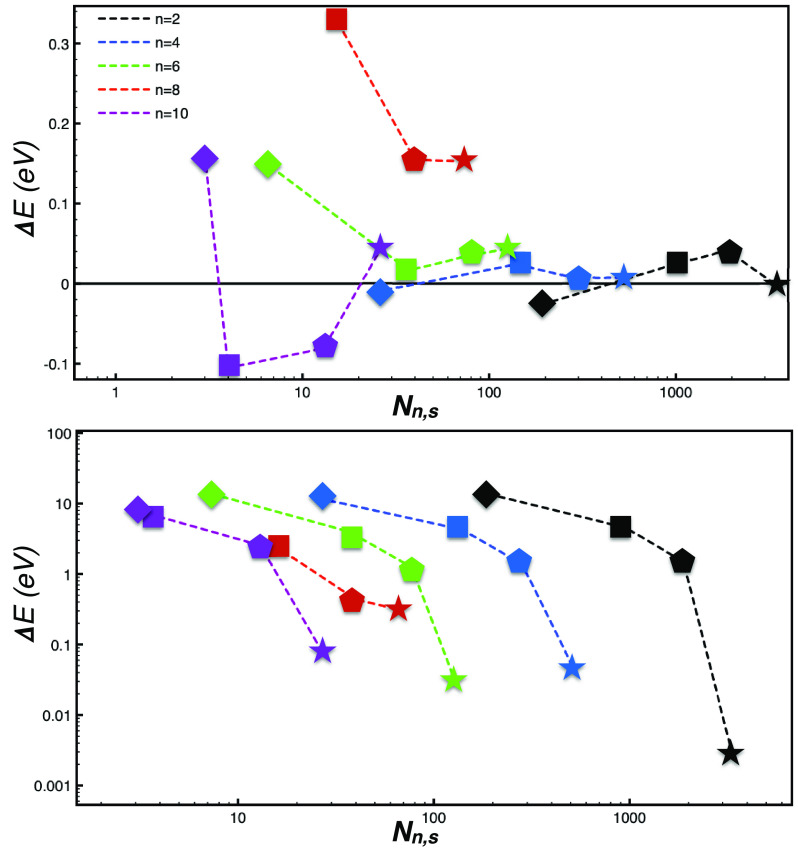
Errors (upper panel) and absolute errors (lower panel) in the band gap of crystalline silicon (see text), which were calculated using eq 11 (upper panel) or eq 8 (lower panel). The number of real-space points *N*_*n*,*s*_ refers to *n* = 2 (black), *n* = 4 (blue), *n* = 6 (green), *n* = 8 (red), and *n* = 10 (green). The threshold *s* was set to 0 (star), 0.5 (pentagon), 1 (square), and 2 (diamond).

Errors relative to the fully converged band gap are displayed in Figure 2. Our method permits us to lower the number of grid points by two orders of magnitude while retaining an accuracy of ca. 20 meV. In contrast, eq 8 always gives errors larger than 100 meV. In this case, these large deviations also obliged us to use a logarithmic scale in the bottom panel of Figure 2. Interestingly, the use of a single grid point chosen according to eq 10 yields a not-so-huge error in the band gap (1 eV), corresponding to a relative error of 22% in the self-energy.

To further check the quality of our implementation, we added to the code the possibility of calculating dielectric constants. For silicon, we found a discrepancy of only 0.0024% in comparison with an analogous calculation done using the DFPT *ph.x* code within the random phase approximation (RPA) that included local fields.

To compare the Si band gap with previous results, we chose a large cubic supercell comprised of 512 atoms, which is roughly equivalent to an ordinary 2 atom fcc primitive cell coupled with a 6 × 6 × 6 *k*-point mesh. In excellent agreement with the result reported in ref (24) (1.20 eV), which was obtained through the analytic continuation scheme and the results in the range 1.16–1.28 eV reported in ref (31), which were obtained from various integration schemes, we found an indirect gap of 1.21 eV. Such a large system was computed on 32 cores (Intel(R) Xeon(R) Platinum 8160 CPU at 2.10 GHz). Each real-space point took 10 h, and 27 points were required as specified by the parameters *s* = 0 and *n* = 10. Thanks to the symmetry of the wave functions, only points in an eight-atoms subcell were chosen. As the *G*_**r**_ functions were calculated using the conjugate gradient algorithm instead of the Krylov subspace one (see SI), we expected to observe significantly faster calculations using the latter option. Additional details regarding our calculation can be found in the SI together with input files.

### Timing and Scaling

Our method prescribes a group of calculations, each of which addresses a different grid point **r**_α_. Then, the *S*_*i*_(**r**_α_;ω) functions are written on the disk for each **r**_α_. Finally, these small files are, typically, collected on a desktop computer and easily processed by a python utility we called *easy_analyser.py*.^28^ This permits us to trivially add a higher parallelization level to that on the plane-waves of the *pw4gww.x* code. Indeed, a main computing job can be divided in several ones, with each one taking care of a subgroup of grid points. This allows for almost linear scaling up to thousands of computing cores.

We illustrate this with the case of a tetraphenylporphyrin (TPP) molecule. The molecules has 78 atoms and 113 doubly occupied valence states. We chose a cubic simulation cell with an edge of 52.9 Bohr, the PBE approximation for the exchange and correlation potentials,^30^ and a cutoff of 45 Ry for defining the plane-waves basis set. Additional details can be found in the SI together with the input files.

**Figure 3:**
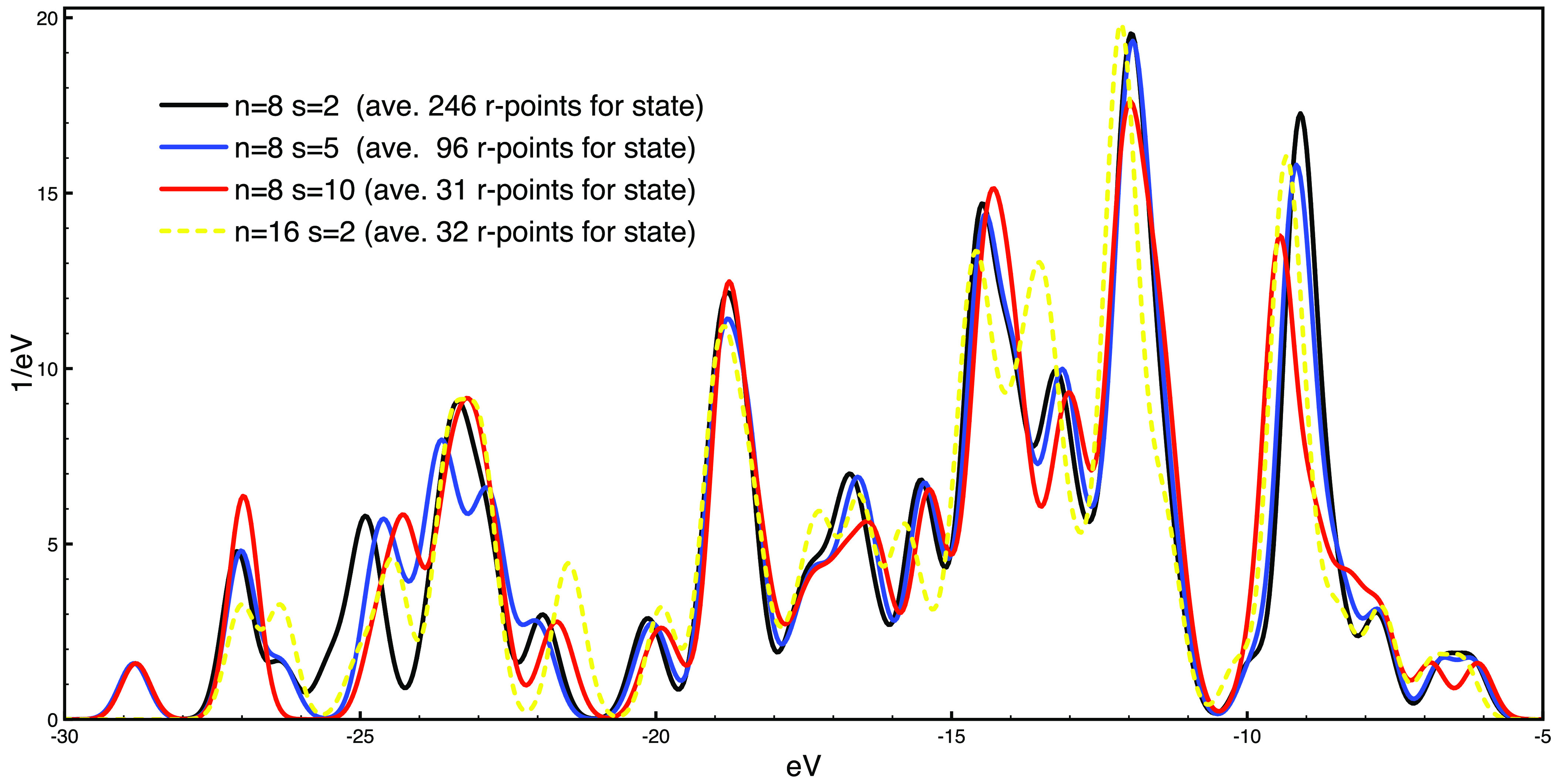
Valence electronic DOS of the TPP molecule calculated at the *G*_0_*W*_0_ level for various values of grid spacing *n* and threshold *s*. The legend reports the average number of grid points that contribute to each orbital. A Gaussian broadening of 0.25 eV was applied.

We performed a *GW* calculation by setting *n* = 8 and *s* = 2. In total, 872 grid points must be processed, and 246 points are then used on average for each orbital. The degree of converge of our calculation can be checked a posteriori by increasing the values of *s* and *n* in the analysis with *easy_analyser.py*. In Figure 3, we show that increasing *s* to 5 au brings almost negligible changes in the valence electronic density of states (DOS) when only 96 points are used on average for each orbital. At the same time, using *n* = 16 gives, in general, only minor changes in the DOS, although only 32 points are used on average for each orbital.

When running on 64 computing cores (IBM POWER9 AC922 at 3.1 GHz^32^), the execution of the code for a single grid point required 271 s. We simultaneously launched 12 sets of calculations while running on 64 × 12 = 768 cores. All the calculations finished after 19 838 s. Hence, going from 64 to 768 cores causes a speed-up of 271 × 872/19838 = 11.9, which is very close to that (12) of for ideal linear scaling.

**Figure 4:**
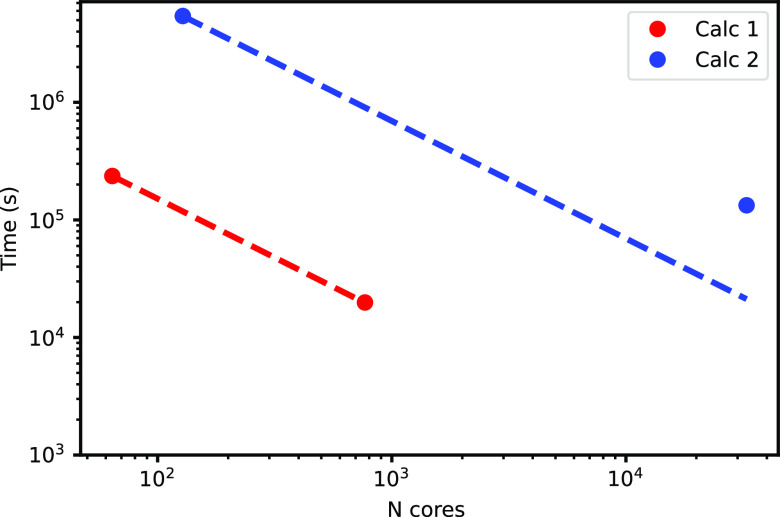
Circles indicate the observed computational time required by the two sets of calculations for TPP as a function of the number of computing cores. The dashed line indicates the ideal linear scaling.

During the development of our code, we had the opportunity to run up to more than 30 000 cores on the same machine. For the same TPP molecule, we calculated a total of 9135 grid points, which were divided on 256 subgroups. For each group, we ran *pw4gww.x* on 128 cores. The computation lasted 594 s lasted for each point. All the calculations were completed after 37 h. This means going from 128 to 128 × 256 = 32768 cores caused a speed-up of (594/3600)*9135/37 = 40.7. This does not fully reach the linear scaling limit (256) only because we experienced some delays in the launch of some groups, as the machine was not entirely available to us. These results are displayed in Figure 4.

**Figure 5:**
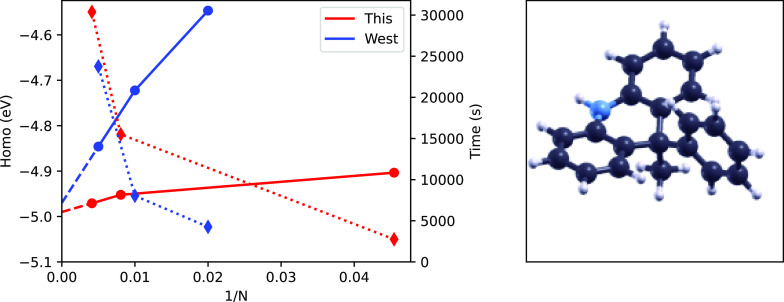
*GW* calculations done with this code (red) and with the WEST code^9^ (blue) for the KuQuinone dye,^33^ which is displayed as a ball-and-stick model (right panel). The HOMO energy is indicated by filled circles (left scale). The total execution time is indicated by filled diamonds (right scale). *N* is the number of grid points (this code) or the number of eigenvectors of the dielectric matrix (WEST code). Solid and dotted lines are guides to the eye. Dashed lines are linear extrapolations.

It is important to show that the performance of our code is at least in-line with those of well established *GW* ones. For this purpose, we compare it with the WEST code,^9^ as it is also implemented in Quantum-Espresso and avoids explicit sums over empty states. We considered the HOMO energy of an organic dye, KuQuinone, that we studied recently^33^ (see Figure 5). The molecule has 38 atoms and 102 valence electrons. We used a cubic simulation cell with an edge of 20 Bohr and norm-conserving pseudopotentials. The energy cutoff defining the plane-wave grid was 70 Ryd. All the input files are available on GitLab.^28^ Tests were carried on a four-core Intel i7 (11th Gen Intel^®^ Core(TM) i7–1165G7) computer. Results are reported in Figure 5. We can see that in the limit of a large *N* value they converge on the same energy within few tens of meV. *N*, in the case of the WEST code, is the number of eigenvectors of the dielectric matrix included in the calculations. In our case *N* is the number of real-space grid points. The corresponding vertical ionization potential can be found by adding −1.85 eV to the HOMO energy to account for the position of the vacuum energy level. Interestingly, our approach, in this particular case, is faster at a comparable level of accuracy. It is worth noting that the large plane-wave cutoff used (70 ryd), which implies a quite dense real-space grid, allowed us to choose *n* = 8.

Indeed, when porting the *n* and *s* parameters from one calculation to another one, *n* can be chosen in such a way that the grid spacing remains fixed. This means taking , where *E*_cutoff_ is the energy cutoff that defines the plane-waves basis set. To port the threshold *s*, we use the convention of Quantum-Espresso, where the modulus squared of a wave function is normalized to the total number of grid points. Hence, we expect , where *V*_cell_ is the volume of the simulation cell and *V*_orbital_ is the volume of the space occupied by the orbital.

Finally, we have to discuss the scaling of the required computational time with respect to the system size. The computational time scales linearly with number of real grid points involved. For orbitals, which exhibit a similar spatial localization, we expect that the required number of grid points is independent of the system size. This means that the time scaling with respect to the system size is the same as that for a single grid point. For this, the most time-consuming and the worst scaling part is the calculation of the screened Coulomb interaction, which scales as the number of valence states *N*_*v*_ times the scaling for applying the KS Hamiltonian operator *N*_*r*,tot_ log(*N*_r,tot_). For the scaling time *T* for a single orbital, we write
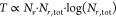
12

If we indicate with *N* the generic system size and assume both *N*_*v*_ ∝ *N* and *N*_*r*,*tot*_ ∝ *N*, the scaling time for a single orbital becomes *N*^2^ log(*N*). We tested eq 12 by estimating the time cost of the calculation for the KuQuinine dye from a calculation for SiH_4_. Inputs file are publicly available.^28^ For this small molecule, we used a cubic simulation cell with an edge of 20 Bohr and energy cutoff-defining planewaves of 25 Ryd. For the KuQuinone dye, we have *N*_*v*_ = 51 and *N*_*r*,tot_ = 108^3^. For SiH_4_, we have *N*_*v*_ = 4 and *N*_*r*,tot_ = 64^3^. A *GW* calculation comprised of 100 grid points for SiH_4_ lasted 220 s, while a calculation comprised of 124 grid points for KuQuinone lasted 15505 s. This is in line with the estimated time length 220 × (124/100) × (108^3^/64^3^) log(108^3^)/ log(64^3^) = 19501 s. As expected, the estimated time is larger than the effective one, as other terms in the calculation of *G* scale more favorable than *W*.

As our approach has been implemented only for the Γ-only sampling of the Brillouin zone, we must adopt supercells for the study of crystalline solids. Luckily, the selection of grid points can be easily limited to the fraction of the supercell that comprises only one primitive cell unit. Hence, the scaling of the computational time will then be *N*^2^ log(*N*), where *N* is the system size. By comparing a corresponding calculation done with *N*_*k*_*k*-points sampling, we identify *N* = *N*_*k*_. To retain the more favorable *N*_*k*_^2^ scaling of ordinary GW codes with *k*-point sampling, an implementation of the evaluation of the KS Hamiltonian that accounts for system periodicity should be added.

## Conclusions

Beside the advantages of our method discussed in the previous sections, additional ones are worth a mention. Our approach can be easily implemented in any DFT-DFPT package. Indeed, the present implementation consists of no more than 5000 Fortran lines. Although it supports, now, only norm-conserving pseudopotentials, the method can be extended to ultrasoft (US)^34^ and projected augmented wave^35^ (PAW) ones. Analogously, it could be extended to support non-collinear spin magnetism and spin–orbit coupling.^36,37^ The support of hybrid density functionals is on the way thanks to their implementation in Quantum-Espresso^38,39^

The code not only scales linearly up to thousands of computing cores but can take advantage of distributed grid computing, as subgroups of real space grid points can be distributed on heterogeneous hardware resources. Luckily, calculations can be refined at a later time by simply adding more grid points. It is interesting to observe that to effectively treat crystalline materials we can retain the present implementation based on the Γ-only sampling of the Brillouin zone and add an appropriate treatment of translational and point symmetries to take real-space grid points only inside the irreducible zone of the primitive cell.

Finally, we make it clear that our method can become very competitive for the study of orbitals localized in a subsystem of the simulation cell (e.g., a molecule in a solvent or a defect in a bulk). Indeed, only grid points localized in a small region of the cell would require calculation.

## Supporting Information Available

Details regarding the calculations of *G*_**r**_ and *W*_**r**_, figures of the HOMO and LUMO of CH_4_, and access to the input files (PDF)
