# Supplementary material for: A Fully Linear Response G0W0 Method That Scales Linearly up to Tens of Thousands of Cores
Source: J Phys Chem A. 2022 May 18;126(21):3384–91. doi: 10.1021/acs.jpca.2c01328 (PMC9169063; doi:10.1021/acs.jpca.2c01328)
Supplement: Supplementary file 1 — jp2c01328_si_001.pdf [file jp2c01328_si_001.pdf]

A Fully Linear Response G_0W_0 Method which Scales Linearly up to Tens of Thousands of Cores

Paolo Umari[*,†,‡]

†*Dipartimento di Fisica e Astronomia, Università di Padova, I-35131 Padova, Italy*

‡*CNR-IOM DEMOCRITOS, Istituto Officina dei Materiali, Consiglio Nazionale delle Ricerche, I-34149 Trieste, Italy*

E-mail: paolo.umari@unipd.it

Calculation of $G_\mathbf{r}$ and $W_\mathbf{r}$

The functions $G_\mathbf{r}$ e $W_\mathbf{r}$ can be calculated using iterative algorithms as in density functional perturbation theory. The $G_\mathbf{r}$ function can be obtained applying to $|\mathbf{r}\rangle$ a shifted and inverted KS Hamiltonian

$$G_\mathbf{r}(\mathbf{r}';\omega) = \left\langle \mathbf{r}' \left| (\hat{H}^{KS} - \epsilon_F - \omega)^{-1} \right| \mathbf{r} \right\rangle \tag{1}$$

where ϵ_F is the Fermi energy which is set half-way the DFT band-gap. In practice, for a given $\mathbf{r}$ we build a Krylov's subspace by means of the $\hat{H}^{KS}$ operator. Then, $\left(\hat{H}_{KS} - \epsilon_F - \omega\right)$ is inverted within that subspace. It's worth noting that the Krylov's subspace does not depend on the frequency ω. Hence, we can calculated $G_\mathbf{r}$ for a set of frequencies ω almost at the same computational cost that for a single one.

We have also implemented the calculation of $G_\mathbf{r}$ using a conjugate gradient algorithm

as it is customary in density functional perturbation theory codes. Although slower, such approach yields fully converged results to be used for testing. For example, the dimension of the Krylov's subspace can be set requiring the results to be the same that from conjugate-gradient.

The calculation of $W_{\mathbf{r}}$ is relatively more complex, we start writing:

$$W_{\mathbf{r}}(\mathbf{r}';\omega) = \left\langle \mathbf{r}' \left| \frac{v\hat{P}\left(\omega\right)}{1 - v\hat{P}\left(\omega\right)} \right| (v\mathbf{r}) \right\rangle \tag{2}$$

with the convention:

$$\langle \mathbf{r}' \left|(v\mathbf{r})\right\rangle = v\left(\mathbf{r}', \mathbf{r}\right) \tag{3}$$

where v is the bare Coulomb potential and $\hat{P}$ is the irreducible polarizability operator. Its action over a generic wave-function Θ can again be calculated through linear response:

$$\hat{P}(\omega)|\Theta\rangle = \sum_v \hat{P}_c \left(\hat{H}^{KS} - \epsilon_v - \omega\right)^{-1} \hat{P}_c \left|(\psi_v \Theta)\right\rangle \tag{4}$$

where the index v runs over the occupied KS orbitals, $\hat{P}_c = \mathbb{1} - \sum_v \left|\psi_v\right\rangle \left\langle\psi_v\right|$ is the projector over the empty KS manifold and where for indicating products in real space of two wave-functions we introduce the notation:

$$\langle \mathbf{r}| \left(\psi_v \Theta\right)\rangle = \psi_v^*\left(\mathbf{r}\right) \Theta\left(\mathbf{r}\right) \tag{5}$$

In practice for each valence state v we create a Krylov's subspace using the seed $\hat{P}_c \left|(\psi_v \mathbf{r})\right\rangle$ and applying the operator $\left(\hat{H}^{KS} - \epsilon_v\right)$. This permits to easily apply the irreducible polarizability operator $\hat{P}$. Then, we follow the iterative Richardson algorithm:

$$\begin{aligned} \left|w_1\left(\omega\right)\right\rangle = & \quad v\hat{P}\left(\omega\right)\left|(v\mathbf{r})\right\rangle \\ \left|w_{n+1}\left(\omega\right)\right\rangle = & \quad \left|w_n\left(\omega\right)\right\rangle - \alpha(1 - v\hat{P}\left(\omega\right)\left|w_n\right\rangle + \alpha v\hat{P}\left(\omega\right)\left|(v\mathbf{r})\right\rangle \end{aligned} \tag{6}$$

where the index n runs over the iterations and α is a small < 1 real parameter. Conver-

gence, defined by a threshold s_w, is reached when $\left| |w_{n+1}(\omega)\rangle - |w_n(\omega)\rangle \right|^2 < s_w$. During the iterations the Krylov's subspaces are augmented in order to represent $\hat{P}$ well for all the wave-functions $|w_n(\omega)\rangle$. The same subspaces are also augmented when the frequency ω is changed. In this way the computational cost for an entire set of frequencies is closed to the one for a single one.

HOMO and LUMO of CH_4

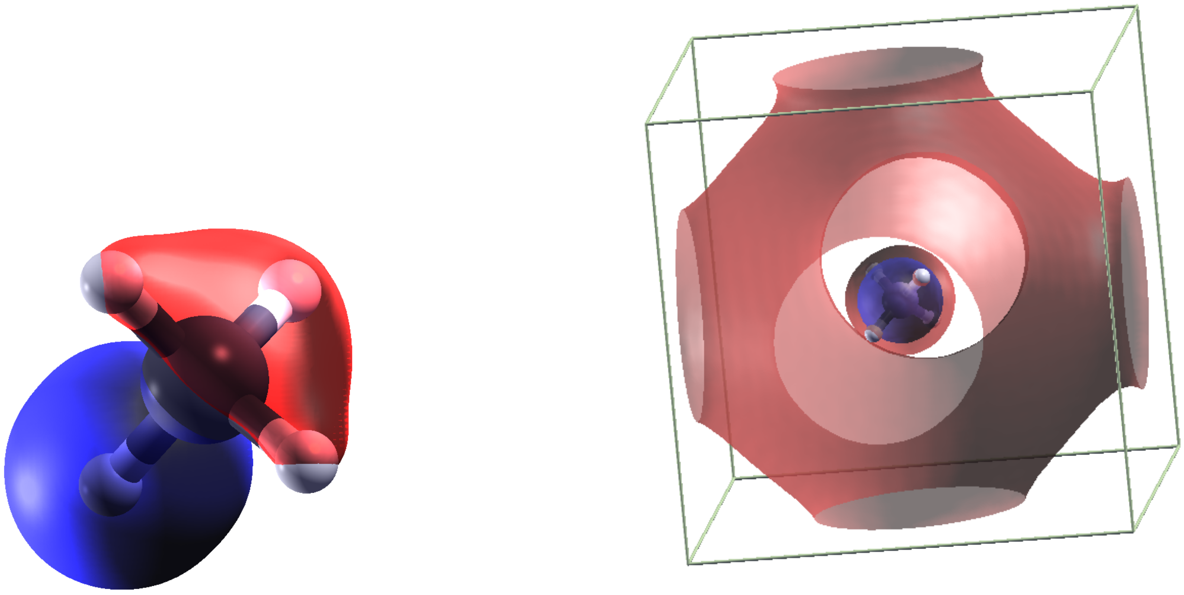

Figure S1: Isosurfaces for the HOMO (left) and LUMO (right) orbitals of the CH_4 molecule. For the LUMO the edges of the periodic simulation cell are also reported

Input Files

All the input files together with the relative pseudopotentials can be found at `https://gitlab.com/paoloumari/easy-gw`
